# Haemodynamic effects of selective venoconstriction with centhaquine in spontaneously breathing patients with mixed hypovolaemic‐vasodilatory shock

**DOI:** 10.1113/EP093743

**Published:** 2026-09-01

**Authors:** Athanasios Chalkias, Aman Khanna, Krish Vaidya, Dharmesh Shah, Anil Gulati

**Affiliations:** ^1^ Institute for Translational Medicine and Therapeutics University of Pennsylvania Perelman School of Medicine Philadelphia Pennsylvania USA; ^2^ Outcomes Research Consortium Houston Texas USA; ^3^ Department of Critical Care Medicine Tzaneio General Hospital Piraeus Greece; ^4^ Aman Hospital and Research Centre Organisation Vadodara Gujarat India; ^5^ I Cure Heart Care Vadodara Gujarat India; ^6^ Pharmazz India Pvt. Ltd. Greater Noida Uttar Pradesh India; ^7^ Pharmazz Inc., Research and Development Willowbrook Illinois USA; ^8^ Department of Bioengineering The University of Illinois at Chicago Chicago Illinois USA; ^9^ Midwestern University Downers Grove Illinois USA

**Keywords:** circulatory dynamics, shock, venoconstriction

## Abstract

Circulatory shock remains difficult to manage because fluid resuscitation and catecholamines may exacerbate vascular dysfunction and tissue hypoperfusion. This prospective physiological analysis, embedded within a multicentre phase IV study, examined whether selectively targeting the venous circulation with centhaquine improves circulatory dynamics in spontaneously breathing patients with mixed hypovolaemic–vasodilatory shock (MHVS). Fifteen spontaneously breathing adults with MHVS received centhaquine (0.01 mg kg^−1^) in addition to standard care. Over 300 min, mean circulatory filling pressure analogue increased from 6.03 ± 0.22 to 7.28 ± 0.21 mmHg and driving pressure for venous return from 5.88 ± 0.22 to 7.16 ± 0.21 mmHg (adjusted *P *< 0.001 for both), while estimated central venous pressure remained near atmospheric pressure and resistance to venous return remained unchanged. Cardiac output increased from 5.62 ± 0.22 to 6.80 ± 0.24 L min^−1^ and mean arterial pressure from 56.8 ± 1.5 to 69.8 ± 2.0 mmHg (adjusted *P *< 0.001 for both). Effective arterial elastance and systemic vascular resistance did not significantly change over time. Patients received 677 ± 79 mL of crystalloids during the study period, and no drug‐related adverse events were observed. The findings of this preliminary physiological study suggest that selective venoconstriction may augment venous return and systemic haemodynamics in spontaneously breathing patients with MHVS without evidence of major adverse haemodynamic effects.

## Abstract (highlights)

- **What is the central question of this study?**
  Does selectively targeting the venous circulation with centhaquine improve circulatory dynamics in spontaneously breathing patients with mixed hypovolaemic–vasodilatory shock (MHVS)?
- **What is the main finding and its importance?**
  Selective venoconstriction may augment venous return and systemic haemodynamics in spontaneously breathing patients with MHVS without evidence of major adverse haemodynamic effects.

## 1 INTRODUCTION

Combined shock is a life‐threatening condition defined by the coexistence of a primary shock state with one or more additional pathophysiological components (Gaieski & Mikkelsen, 2025). Among its variants, mixed hypovolaemic–vasodilatory shock (MHVS) represents a particularly complex and clinically relevant phenotype frequently encountered in perioperative, emergency and critical care settings. The pathophysiology of MHVS is typically biphasic, beginning with an early hypovolaemic phase driven by absolute or relative intravascular volume depletion, followed by progressive pathological vasodilation, capillary leak and vascular hyporesponsiveness. This interaction establishes a haemodynamic milieu prone to endothelial stress, disruption of microcirculatory flow and downstream organ injury (Khorsand et al., 2023).

During the initial phase of MHVS, fluid resuscitation may transiently improve macrohaemodynamics by reducing vascular capacitance and/or resistance to venous return (RVR). However, as vasodilation becomes predominant, intravascular fluid is progressively maldistributed, capillary leak further reduces effective circulating volume, and excessive fluid administration promotes third‐spacing and tissue oedema (Madanayake et al., 2021; Magder, 2016; Walsh et al., 2023). Catecholamines are therefore typically reserved for refractory hypotension; nonetheless, they may exacerbate microvascular flow disturbances and tissue hypoperfusion and are associated with multiple adverse effects owing to the widespread distribution of adrenergic receptors (Chioléro et al., 1991; Gelman, 2020; Hartmann et al., 2017; Rossaint et al., 2023). Notwithstanding these limitations, contemporary shock management remains predominantly arterial‐centric, despite robust physiological evidence identifying venous capacitance and stressed volume as the principal determinants of venous return and cardiac output (CO).

We have previously demonstrated that selectively targeting the venous circulation with centhaquine citrate improves haemodynamics in haemorrhagic shock while avoiding several deleterious effects associated with conventional vasopressors (Chalkias et al., 2024; Gulati, Choudhuri et al., 2021; Gulati, Jain et al., 2021). Centhaquine preferentially activates α_2B_‐adrenoreceptors, which are abundant in veins but sparse in arteries, resulting in selective venoconstriction, while concurrent activation of central α_2A_‐adrenoreceptors attenuates sympathetic outflow and reduces systemic vascular resistance (SVR) (Rutlen et al., 1981). By augmenting venous return without excessive arterial constriction, early centhaquine administration may optimise preload and improve CO in MHVS, particularly during the transition from hypovolaemia to vasodilation when fluid management becomes increasingly challenging.

Accordingly, the aim of this physiological study was to investigate whether selectively targeting the venous circulation with centhaquine improves circulatory dynamics in spontaneously breathing patients with MHVS.

## 2 METHODS

### 2.1 Ethics approval

The study was conducted from 5 June 2023 to 22 March, 2025, in compliance with the International Council for Harmonisation Guideline for Good Clinical Practice (ICH‐GCP), the *Declaration of Helsinki*, and all applicable local regulatory requirements. The study protocol (PMZ‐2010/CT‐4.1/2019, version 1.0 dated 16 October 2019) received approval from the Drugs Controller General of India (DCGI), under the Directorate General of Health Services, Ministry of Health & Family Welfare, Government of India (DCGI CT NOC No.: CT/ND/110/2020). Ethical approval for this study was provided by the Institutional Ethics Committee of Aman Hospital and Research Center, Vadodara, India (ECR/857/Inst/GJ/2016/RR‐19; 26 November 2022). Prior to the commencement of patient enrolment, the study protocol received approval from the ethics committee at each participating institution. The trial was registered in the Clinical Trials Registry‐India (CTRI/2021/01/030263), the United States National Library of Medicine, and at ClinicalTrials.gov (NCT05956418).

### 2.2 Study design

The study was designed at the outset of data collection for the ‘Study to Assess the Safety and Efficacy of Centhaquine in Hypovolemic Shock Patients’ (PMZ‐2010/CT‐4.1/2019; ClinicalTrials.gov Registration: NCT05956418), an ongoing prospective, multicentre, open‐label, single‐arm, phase IV clinical study to evaluate the safety and efficacy of the first‐in‐class drug centhaquine citrate (LYFAQUIN™) in adults with hypovolaemic shock across 17 healthcare institutions.

### 2.3 Objective

The primary objective was to characterise temporal changes in integrated circulatory dynamics following early selective venoconstriction with centhaquine in spontaneously breathing patients with MHVS. Haemodynamic assessment incorporated venous return physiology, cardiac performance, arterial load and circulatory energetics, with particular emphasis on mean circulatory filling pressure analogue (*P*_mca_) and the driving pressure for venous return (VRdP) as central determinants of venous return.

### 2.4 Patient eligibility

We included neurologically intact, spontaneously breathing patients who were randomly selected from those enrolled in the underlying phase IV study. Patient selection was performed using simple randomisation by an independent individual with no involvement in the study. Individuals were eligible if they met the following criteria: (i) were adults (≥18 years) with MHVS; (ii) presented to the emergency department or intensive care unit with a systolic arterial pressure (SAP) of 90 mmHg or lower; (iii) were receiving standard treatment for shock; and (iv) had a blood lactate level >2.0 mmol L^−1^.

Individuals were excluded if they had any terminal illness unrelated to MHVS, altered consciousness, known pregnancy, cardiac arrest before enrolment, a do‐not‐resuscitate order, participation in another interventional study, or preexisting systemic diseases (including cancer, chronic kidney disease, liver failure, pulmonary hypertension, structural heart disease or decompensated heart failure).

Eligible patients were approached by a physician who provided a verbal explanation of the study. Those who agreed to participate provided written informed consent using the provided consent form. Informed consent was obtained from all participants and/or their legal guardians. All patients received care consistent with institutional protocols and standard clinical practice for the management of circulatory shock, including appropriate assessment, resuscitation, and supportive treatment.

### 2.5 Intervention

After initial screening, eligible patients received an intravenous infusion of 0.01 mg kg^−^^1^ centhaquine diluted in 100 mL of normal saline and administered over a period of 60 min, in addition to standard care. Administration of fluid resuscitation with crystalloids was left to the discretion of the attending physicians. No patient received noradrenaline, any other vasopressor agents or mechanical ventilation during the study observation period.

### 2.6 Measurements

#### 2.6.1 Arterial blood pressure

Arterial blood pressure was monitored non‐invasively with a contemporary automated oscillometric cuff manometer placed on the upper arm, following standard clinical procedures, at baseline, every hour during the first 48 h, and additionally as required (Chatterjee et al., 2010; Lakhal et al., 2018). Systolic arterial pressure, diastolic arterial pressure (DAP) and mean arterial pressure (MAP) were recorded. All measurements were obtained with the patient in a supine position, using the same device and cuff size for each individual throughout the study to ensure internal consistency. Arterial pressure measurements were used to assess temporal changes in arterial loading conditions and were interpreted in conjunction with echocardiographic and venous return‐related variables rather than as standalone determinants of circulatory status.

#### 2.6.2 Rationale for estimated central venous pressure over direct catheterisation

Direct central venous catheterisation was not performed in this study to avoid unnecessary risks associated with invasive procedures in spontaneously breathing, non‐intubated patients who were managed with non‐invasive monitoring. From a physiological perspective, the primary objective of central venous pressure (CVP) assessment in this study was not to isolate an instantaneous right atrial pressure at a specific point in the respiratory cycle, but to establish the effective downstream sink pressure required for integrated Guytonian systemic circuit analysis. Estimating CVP from contemporaneously acquired haemodynamic variables provides an internally consistent representation of the underlying circulatory state governing venous return and is well suited for deriving physiologically integrated variables (Maas et al., 2012; Parkin & Leaning, 2008).

#### 2.6.3 Mathematical estimation of CVP

To address these procedural limitations and potential physiological artifacts, CVP was mathematically derived using a validated haemodynamic analogue of Ohm's law for the systemic circulation, which integrates MAP, CO, and SVR. CVP was calculated via the following rearranged equation (Chan et al., 2014; De Backer et al., 2011; Oh et al., 2021; Secomb, 2016; Sun et al., 2021): CVP = MAP − [(SVR × CO)/80].

In spontaneously breathing hypovolaemic patients, low or near‐atmospheric right atrial pressures are physiologically plausible due to reduced stressed volume, preserved inspiratory pressure swings, and low intrathoracic pressures (Magder, 2015, 2016, 2018). By averaging estimated CVP values across repeated measurements, this approach minimised procedural and respiratory sources of variability associated with direct catheterisation, although small transient physiological fluctuations were not fully captured.

#### 2.6.4 Echocardiography

Transthoracic echocardiography was conducted at baseline (0 min) and at 60, 120 and 300 min following centhaquine administration by an experienced echocardiographer who remained blinded to the participant identity and study timeline. Measurements included left ventricular ejection fraction, left ventricular outflow tract (LVOT) diameter, LVOT velocity time integral (LVOT‐VTI), stroke volume and CO (Kumar et al., 2004; Magder, 2015). Each echocardiographic parameter was determined by averaging five measurements, irrespective of the respiratory phase, and analysed retrospectively.

SVR was estimated non‐invasively using Doppler echocardiography through the ratio of mitral regurgitant velocity to LVOT‐VTI (MRV/LVOT‐VTI), a validated surrogate of SVR (Abbas et al., 2004). American Society of Echocardiography Guidelines support echocardiographic estimation of SVR in critically ill patients (Porter et al., 2015), and this approach has recently been prospectively validated against transpulmonary thermodilution‐derived SVR index measurements (Gaubert et al., 2020).

#### 2.6.5 Determinants of venous return

The algorithm for estimating *P*_mca_ and related venous return parameters has been described (Chalkias, Laou et al., 2022; Chalkias, Laou, Papagiannakis et al., 2022; Chalkias, Laou, Papagiannakis, Varvarousi et al., 2022; Parkin & Leaning, 2008) and validated (Persichini et al., 2022; Werner‐Moller et al., 2022; Wijnberge et al., 2018) in previous studies. In summary, grounded in Guyton's representation of the systemic circulation – where CO equals venous return [CO = VR = (*P*_mcf_ − CVP)/RVR, where *P*_mcf_ is mean circulatory filling pressure] – *P*_mca_ can be calculated using the equation *P*_mca_ = (*a* × CVP) + (*b* × MAP) + (*c* × CO). In this expression, *a* and *b* are dimensionless coefficients that sum to unity. By assuming a venous‐to‐arterial compliance ratio of 24:1, *a* is set to 0.96 and *b* to 0.04, corresponding to the relative influence of the venous and arterial compartments. The coefficient *c* represents a composite of the venous–arterial compliance ratio (0.96) and venous resistance (SVR × 0.038), has units of resistance, and is calculated using a formula that incorporates patient age, height and weight (Moller & Parkin, 2022; Wijnberge et al., 2018):

$$c=\frac{0.96\times 0.038\times (94.17+0.193\times [\text{age in years}])}{4.5\times 0.99^{\left([\text{age in years}]-15\right)}\times 0.007184\times {\left[\text{height in cm}\right]}^{0.725}\times {\left[\text{weight in kg}\right]}^{0.425}}$$

Additionally, the VRdP (VRdP = *P*_mca_ − CVP) and the resistance to venous return [RVR = (*P*_mca_ − CVP)/CO] were calculated. The latter equation describes venous return under transient haemodynamic imbalances, with *P*_mca_ representing the average circulatory pressure and RVR reflecting the resistance encountered by blood returning to the heart (Berger, Moller et al., 2016; Berger, Moller, Weber et al., 2016).

#### 2.6.6 Efficiency measures

Heart efficiency (*E*_h_) was defined as the ratio of the pressure difference between *P*_mca_ and CVP to *P*_mca_, expressed as *E*_h_ = (*P*_mca_ − CVP)/*P*_mca_, with values ranging from 0 to 1. This formula serves as an index of cardiac performance, indicating how effectively the heart manages the VRdP relative to *P*_mca_ and CVP (Chalkias, Laou et al., 2022; Chalkias, Laou, Papagiannakis et al., 2022; Chalkias, Laou, Papagiannakis, Varvarousi et al., 2022). An *E*_h_ value approaching 1 indicates normal cardiac function with CVP near zero. During cardiac arrest or cessation of ejection, right atrial pressure (CVP) equals *P*_mca_, causing *E*_h_ to approach zero (Chalkias, Laou, Papagiannakis et al., 2022).

Cardiac power was calculated as Power = CO × (MAP − CVP) × 0.0022, while cardiac power output (CPO) was determined by CPO = (CO × MAP)/451. Cardiac power quantifies the rate at which the heart delivers energy to the systemic circulation at the aortic root to sustain perfusion of vital organs, particularly under shock conditions (Fincke et al., 2004). Power efficiency (*E*_power_) was defined as the ratio of the change in power to the corresponding change in *P*_mca_: *E*_power_ = Δ(CO × (MAP − CVP) × 0.0022)/Δ*P*_mca_. Unlike *E*_h_, which is a static measure, *E*_power_ reflects dynamic changes in cardiac power relative to variations in *P*_mca_ (Sondergaard et al., 2019). Cardiac power indices were included because they integrate both flow and pressure generation and may therefore better characterise circulatory energetics during shock than CO alone.

Volume efficiency (*E*_vol_) was calculated as *E*_vol_ = Δ(*P*_mca_ − CVP)/Δ*P*_mca_, with values ranging from 0 to 1. This dynamic parameter represents how effectively interventions such as fluid administration, vasopressors or inotropes enhance VRdP in relation to *P*_mca_, thereby improving CO and oxygen delivery (Sondergaard et al., 2019).

#### 2.6.7 Other circuit parameters

Other haemodynamic parameters were also assessed, including venous compartment resistance (*R*_ven_ = SVR × 0.038), arterial resistance (*R*_art_ = MAP/CO), arterial compliance [*C*_art_ = SV/(SAP − DAP)], and effective arterial elastance (*E*_a_ = MAP/SV). The latter serves as an integrative measure of cardiac afterload, capturing both steady and pulsatile contributions.

### 2.7 Data collection, monitoring, and safety evaluation

Data analysis was based on predefined and contemporaneously recorded measurements. Clinical information was collected by examining patient charts and electronic medical records. Data collection included demographic and morphometric data, clinical parameters, chest X‐rays, electrocardiograms, blood counts and chemistry results. Respiratory function (oximetry; breathing frequency, pattern and effort) was also evaluated (Scott & Kaur, 2020). Each patient was continuously monitored throughout hospitalisation until discharge. Remote monitoring was conducted to identify early abnormal patterns, inconsistencies, credibility issues and other anomalies. The multivariate imputation by chained equations (MICE) method was used to address any missing data. Protocol deviations, used to assess study feasibility, were defined as any departure from the study protocol. When necessary, the attending physician was asked to provide justification for the deviation. The study investigators assessed safety by monitoring adverse events, conducting physical examinations, evaluating vital signs, reviewing electrocardiograms and examining other clinical parameters. Any adverse events that appeared or worsened during or following centhaquine treatment were carefully documented and classified according to system organ class and preferred term, using the most recent version of the International Council for Harmonisation (ICH) Medical Dictionary for Regulatory Activities (MedDRA).

### 2.8 Statistical analysis

Statistical analysis was performed using R version 4.3 and GraphPad Prism version 10.1.2 (GraphPad Software, Boston, MA, USA). Continuous variables are presented as mean ± standard deviation (SD) or median (interquartile range [IQR]), as appropriate. Differences between time points were assessed using repeated‐measures ANOVA. The Benjamini–Hochberg correction was applied to adjust *P*‐values, and adjusted *P*‐values less than 0.05 were considered statistically significant.

## 3 RESULTS

Fifteen participants [12 (80%) men] were included. The median age was 40 (28–48.5) years, and the median body mass index was 24 (23–25) kg m^−2^. Medical histories were notable only for respiratory disease [1 (6.7%)] and hepatitis [6 (40%)]. Causes of MHVS included gastroenteritis (vomiting, abdominal pain, and/or diarrhoea) [5 (33.3%)], dengue fever [2 (13.3%)], acute appendicitis [1 (6.7%)], enteric fever with gastroenteritis [4 (26.7%)], falciparum malaria [1 (6.7%)], lower respiratory tract infection with severe vomiting [1 (6.7%)] and acute pyelonephritis with severe vomiting [1 (6.7%)].

The predominant physiological component was hypovolaemia. Nine (60%) patients presented with class III shock and six (40%) with class IV shock. At presentation, mean shock index was 1.26 ± 0.28, blood lactate 2.6 ± 0.3 mmol L^−1^, base deficit −2.25 ± 1.6 mmol L^−1^, pH 7.23 ± 0.04, $P_{{\mathrm{aO}}_{2}}$/$F_{{\mathrm{iO}}_{2}}$ ratio 365 ± 30, haemoglobin 12 ± 1.45 g dL^−1^ and creatinine 1.1 ± 0.46 mg dL^−1^. Over the 300‐min study period, patients received an average of 677 ± 79 mL of crystalloids. No significant changes in respiratory function were observed during this period.

### 3.1 Determinants of venous return

Administration of centhaquine increased *P*_mca_ (adjusted *P *< 0.001) and VRdP (adjusted *P *< 0.001) over the study period (Figure 1). Estimated CVP values remained low and near atmospheric pressure throughout the study period without significant temporal variation (adjusted *P* = 0.992; Figure 2). Resistance to venous retVR did not vary significantly (adjusted *P* = 0.861; Figure 2), while *E*_vol_ demonstrated a modest but statistically significant change over time (adjusted *P* = 0.043; Figure 3).

**FIGURE 1:**
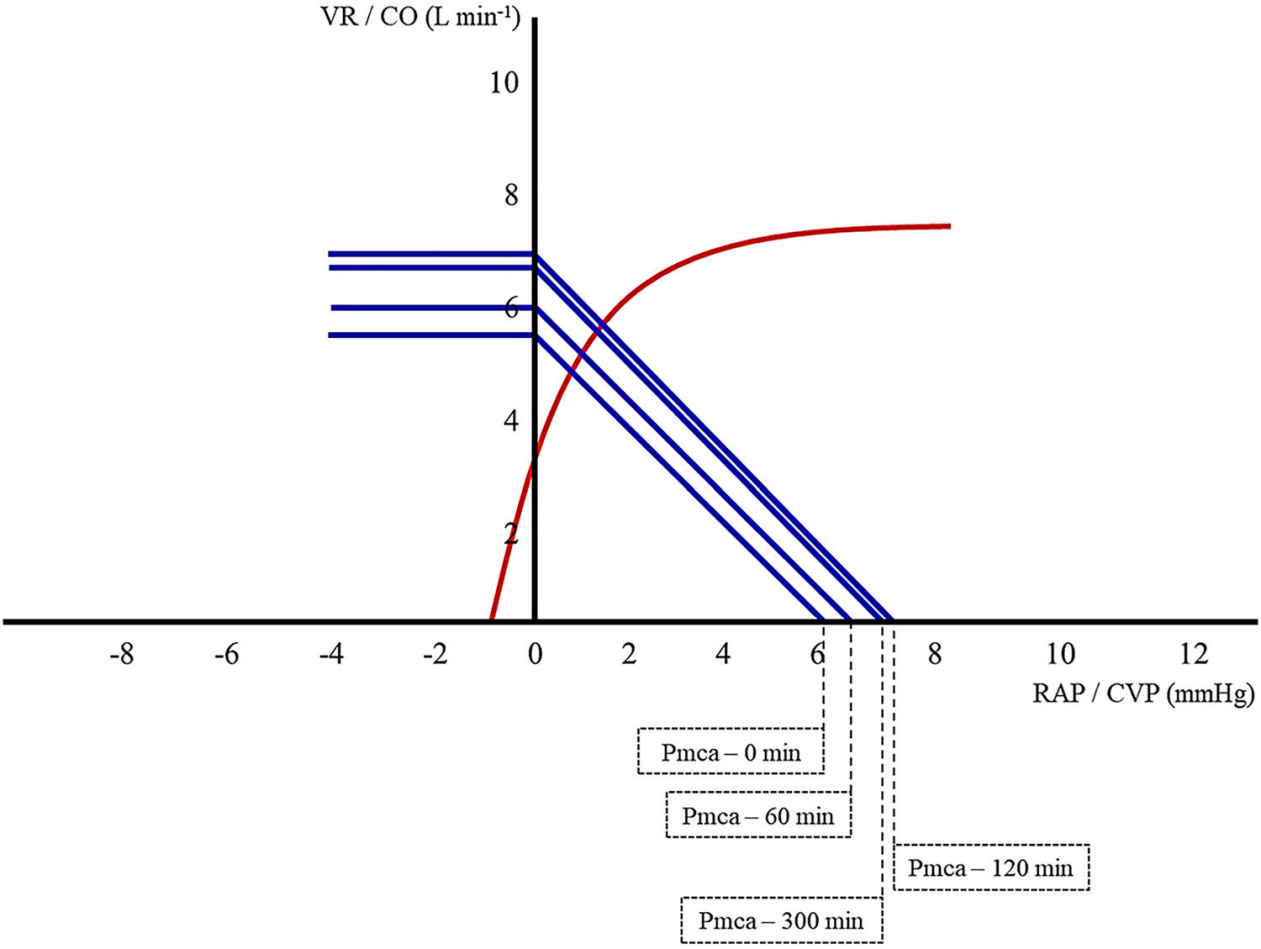
Graphical representation of the interplay between venous return (blue lines) and cardiac function (red line) in spontaneously breathing patients with MHVS. Administration of centhaquine increased the gradient between *P*_mca_ and right atrial pressure (CVP), without altering RVR or cardiac function. This shift moves the equilibrium point toward the upper right, resulting in enhanced venous return and cardiac output. The cardiac function curve begins in the negative range because pleural pressure surrounds the heart, causing intrachamber pressure to be negative relative to atmospheric pressure when the heart is empty. CO, cardiac output; CVP, central venous pressure; *P*_mca_, mean circulatory filling pressure analogue; RAP, right atrial pressure; VR, venous return.

**FIGURE 2:**
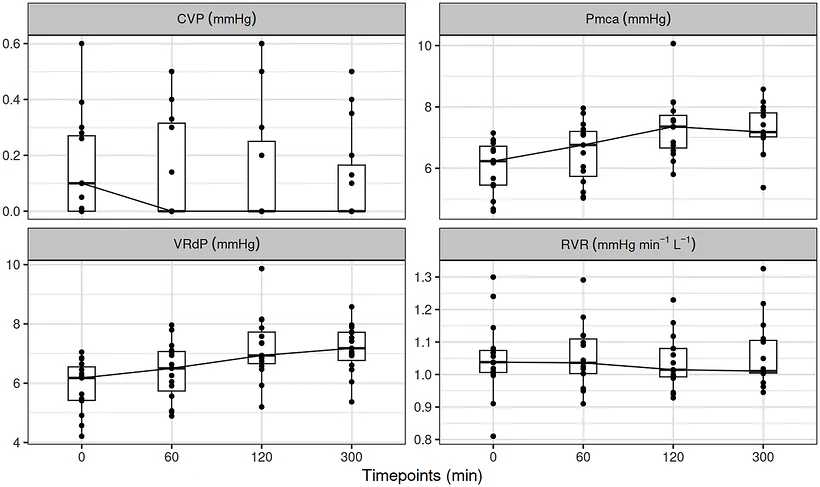
Variations in *P*_mca_ and other determinants of venous return. CVP, central venous pressure; *P*_mca_, mean circulatory filling pressure analogue; RVR, resistance to venous return; VRdP, venous return driving pressure.

**FIGURE 3:**
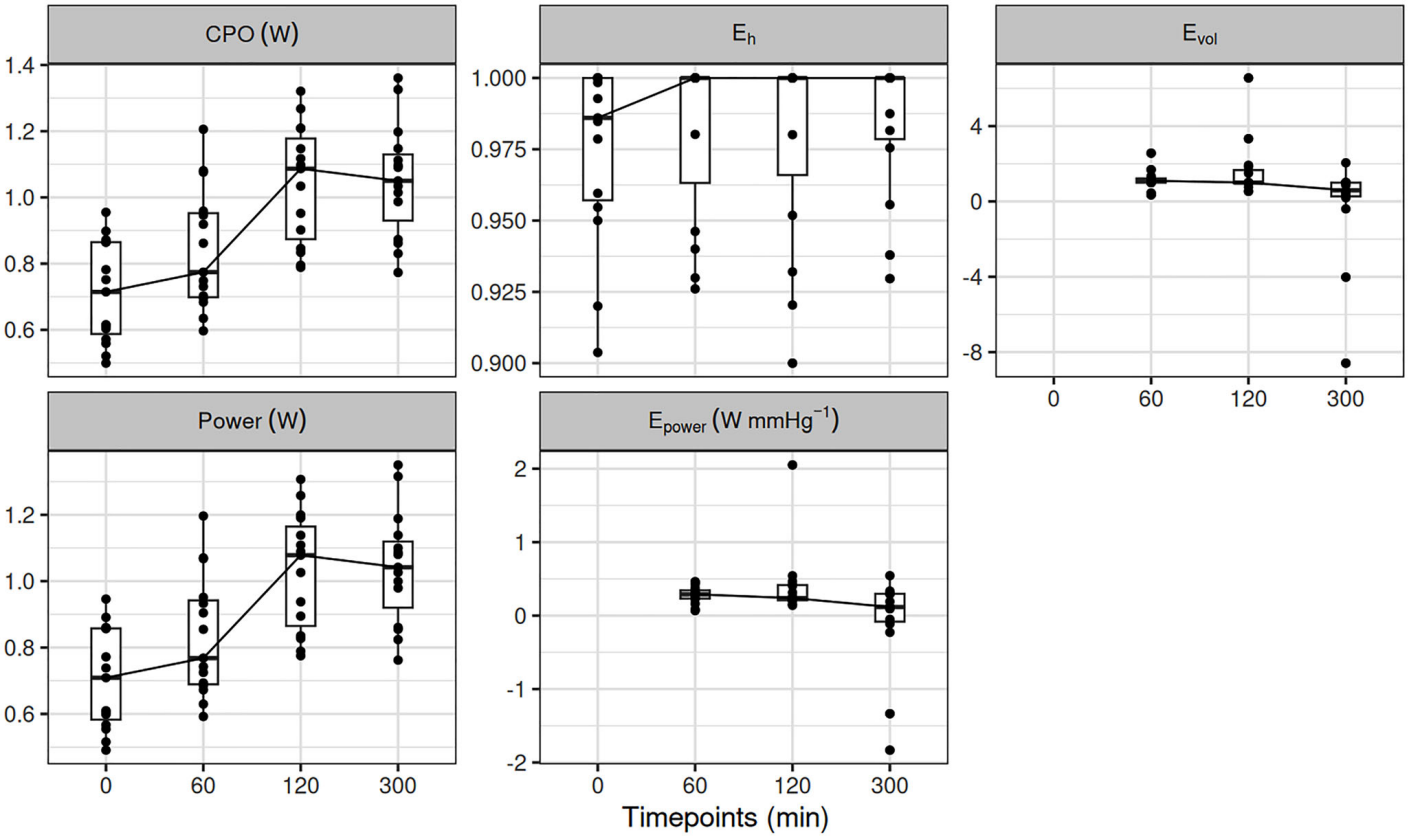
The impact of centhaquine on cardiac performance and its effectiveness in raising VRdP in relation to an increase in *P*_mca_. CPO, cardiac power output; *E*_h_, efficiency of the heart; *E*_power_, power efficiency; *E*_vol_, volume efficiency.

### 3.2 Venous and arterial parameters

Venous compartment resistance remained stable throughout the study period (adjusted *P =* 0.861; Figure 4). Arterial compliance exhibited a modest increase during the first 120 min following centhaquine administration, which became more pronounced thereafter (adjusted *P *< 0.001). In contrast, *R*_art_, *E*_a_ and SVR did not change significantly over time (adjusted *P =* 0.992, 0.096 and 0.861, respectively). Despite the absence of significant changes in resistance‐related indices, MAP increased significantly across time points (adjusted *P <* 0.001; Figure 5).

**FIGURE 4:**
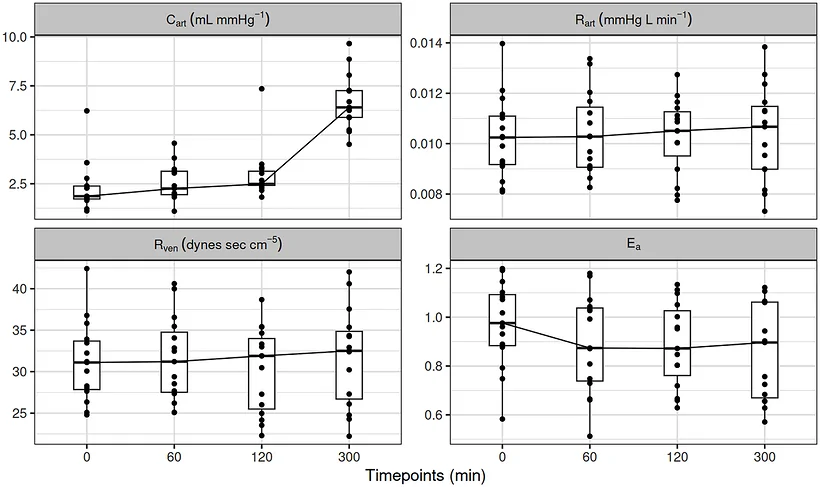
Changes in venous and arterial compartment parameters. *C*_art_, arterial compliance; *E*_a_, effective arterial elastance; *R*_art_, arterial resistance; *R*_ven_, venous compartment resistance.

**FIGURE 5:**
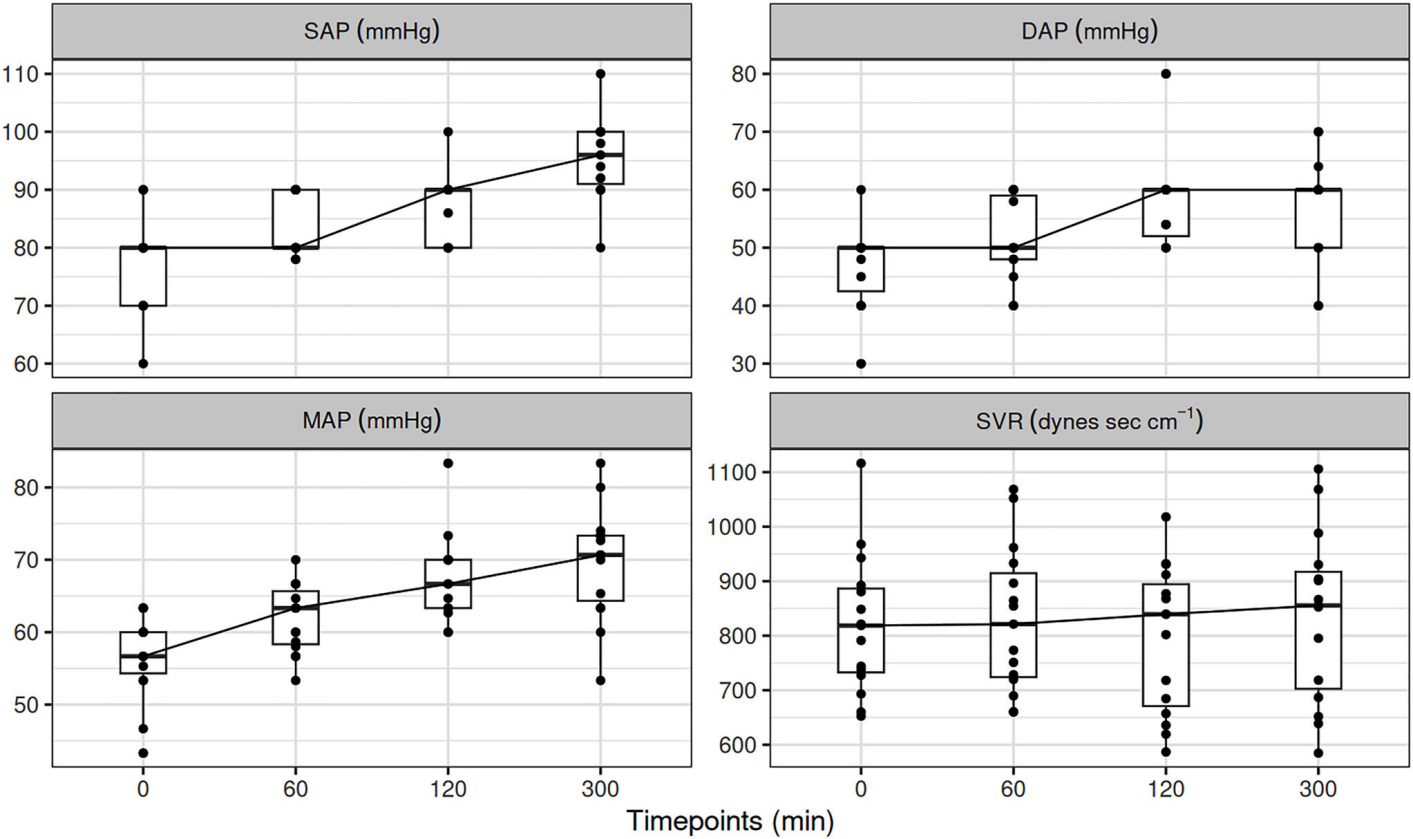
Variations in arterial blood pressure and systemic vascular resistance. DAP, diastolic arterial pressure; MAP, mean arterial pressure; SAP, systolic arterial pressure; SVR, systemic vascular resistance.

### 3.3 Cardiac performance and echocardiography

The inferior vena cava diameter increased during the first 120 min following centhaquine administration and subsequently plateaued over the remaining 180 min (adjusted *P <* 0.001; Table 1). LVOT‐VTI increased consistently at all post‐treatment time points (adjusted *P <* 0.001), whereas other LVOT geometric and functional parameters remained stable (Figure 6). These changes were accompanied by parallel increases in CO (adjusted *P <* 0.001), power (adjusted *P <* 0.001) and CPO (adjusted *P <* 0.001), without a significant change in *E*_h_ (adjusted *P =* 0.917). Power efficiency increased significantly during the first 120 min, followed by a gradual decline over the subsequent 180 min, while remaining above baseline values (adjusted *P =* 0.026).

**TABLE 1:** Haemodynamic variables before and after centhaquine administration.

	0 min^#^	60 min	120 min	300 min	Adjusted *P*
*P*_mca_ (mmHg)	6.03 (0.22)	6.53 (0.26)	7.34 (0.26)	7.28 (0.21)	<0.001
VRdP (mmHg)	5.88 (0.22)	6.40 (0.25)	7.19 (0.28)	7.16 (0.21)	<0.001
RVR (mmHg min^−1^ L^−1^)	1.05 (0.03)	1.06 (0.03)	1.04 (0.02)	1.06 (0.03)	0.861
IVC (cm)	0.97 (0.04)	1.10 (0.03)	1.18 (0.03)	1.19 (0.04)	<0.001
CVP (mmHg)	0.15 (0.18)^†^	0.14 (0.20)^†^	0.15 (0.24)^†^	0.11 (0.17)^†^	0.992
LVOT‐VTI (cm)	17.93 (0.96)	20.88 (1.03)	22.99 (0.89)	23.90 (0.84)	<0.001
Power (W)	0.70 (0.04)	0.83 (0.05)	1.03 (0.05)	1.04 (0.04)	<0.001
CO (L min^−1^)	5.62 (0.22)	6.10 (0.27)	6.94 (0.27)	6.80 (0.24)	<0.001
CPO (W)	0.71 (0.04)	0.84 (0.05)	1.04 (0.05)	1.05 (0.04)	<0.001
*E*_h_	0.98 (0.01)	0.98 (0.01)	0.98 (0.01)	0.98 (0.01)	0.917
*E*_power_ (W mmHg^−1^)	NA	0.29 (0.03)	0.41 (0.12)	0 (0.17)	0.026
*C*_art_ (mL mmHg^−1^)	2.32 (0.32)	2.56 (0.23)	2.95 (0.34)	6.72 (0.36)	<0.001
*E*_a_	0.97 (0.04)	0.89 (0.05)	0.89 (0.04)	0.86 (0.05)	0.096
SVR (dyn s cm^−5^)	819.68 (32.8)	829 (34.48)	794.71 (34.72)	836.56 (40.4)	0.861
SAP (mmHg)	76.67 (2.11)	83.07 (1.32)	87.07 (1.52)	95.47 (1.79)	<0.001
DAP (mmHg)	46.87 (1.8)	51.27 (1.79)	57.87 (1.96)	56.93 (2.37)	<0.001
MAP (mmHg)	56.80 (1.51)	61.87 (1.21)	67.60 (1.54)	69.78 (1.98)	<0.001
*E*_vol_	NA	1.11 (0.14)	1.59 (0.4)	−0.20 (0.69)	0.043
*R*_ven_ (dyn s cm^−5^)	31.15 (1.25)	31.50 (1.31)	30.20 (1.32)	31.79 (1.54)	0.861
*R*_art_ (mmHg L min^−1^)	0.01 (0)	0.01 (0)	0.01 (0)	0.01 (0)	0.992

Values are means (SD). ^#^Baseline (before centhaquine administration). ^†^Near atmospheric pressure.

Abbreviations: *C*_art_, arterial compliance; CO, cardiac output; CPO, cardiac power output; CVP, central venous pressure; DAP, diastolic arterial pressure; *E*_a_, effective arterial elastance; *E*_h_, efficiency of the heart; *E*_power_, power efficiency; *E*_vol_, volume efficiency; IVC, inferior vena cava; LVOT‐VTI, left ventricular outflow tract velocity time integral; MAP, mean arterial pressure; *P*_mca_, mean circulatory filling pressure analogue; *R*_art_, arterial resistance; *R*_ven_, venous compartment resistance; RVR, resistance to venous return; SAP, systolic arterial pressure; SVR, systemic vascular resistance; VRdP, driving pressure for venous return.

**FIGURE 6:**
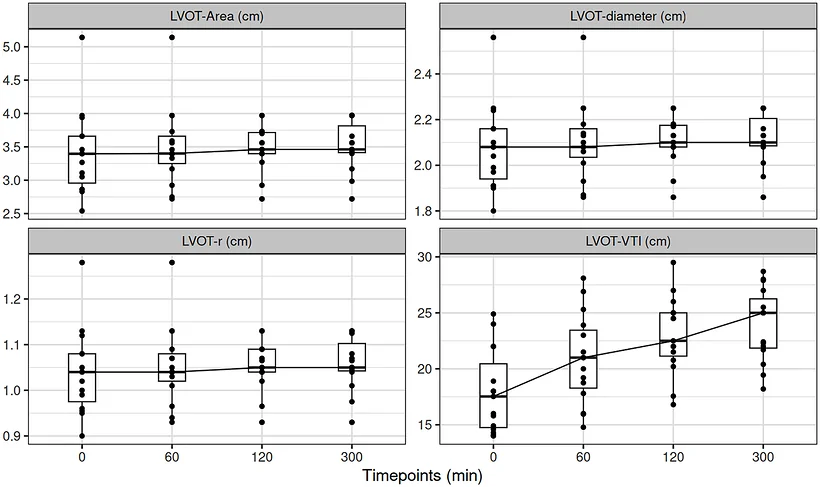
The left ventricular outflow tract and aortic valve were examined using a parasternal long‐axis view. At each post−treatment time point, an increase in LVOT‐VTI relative to baseline was observed. LVOT‐VTI, left ventricular outflow tract velocity time integral.

### 3.4 Data collection, monitoring, and safety evaluation

No protocol modifications, deviations, withdrawals or subject exclusions occurred during the study period. No adverse events judged to be related to centhaquine were observed, and the intervention was well tolerated.

## 4 DISCUSSION

Analysis of this physiological study, nested within a multicentre, open‐label, phase IV clinical study, showed that centhaquine administration was associated with significant increases in *P*_mca_ and VRdP without detectable changes in RVR or CVP. These changes were accompanied by parallel increases in CO, power, CPO and *E*_power_, while *E*_h_ remained unchanged, consistent with preferential preload recruitment. Effective arterial elastance remained stable, whereas *C*_art_ and MAP increased significantly. Selective venoconstriction with centhaquine was well tolerated, with no drug‐related adverse events observed during the study period.

Managing patients with MHVS is inherently challenging because of complex underlying physiological disturbances and heterogeneous clinical presentations. Fluid resuscitation remains a cornerstone of therapy; however, excessive volume administration may increase the risk of adverse events and does not invariably correct the fundamental haemodynamic abnormalities. Such limitations are more likely when cardiovascular function is incompletely characterised at the bedside, potentially leading to suboptimal haemodynamic management. In clinical practice, resuscitation strategies often prioritise arterial pressure‐based targets, whereas the venous circulation – despite its central role in determining venous return, stressed volume and CO – may be comparatively underappreciated.

In the present analysis, centhaquine was associated with favourable changes in circulatory dynamics and a reduction in fluid requirements, consistent with prior preclinical observations (Chalkias et al., 2024; Papapanagiotou et al., 2016). The stability of *E*_h_, alongside parallel increases in CO, power, CPO and *E*_power_, suggests that the observed haemodynamic changes were predominantly attributable to centhaquine‐mediated recruitment of unstressed venous volume and augmentation of venous return, rather than to enhanced intrinsic cardiac contractility. Importantly, the modest fluid administration rate (∼135 mL h^−1^) over the 300‐min observation period makes a substantial contribution of infused volume to these effects unlikely. These findings may have particular relevance during the hypovolaemia‐dominant phase of MHVS, a context in which additional α‐adrenergic receptor activation – such as that induced by conventional vasopressors – can exacerbate hepatic venous constriction, impair splanchnic venous outflow and promote blood sequestration within the splanchnic circulation (Laou et al., 2023; Rothe et al., 1990). By contrast, centhaquine appears to exert minimal constrictive effects on hepatic veins relative to other adrenergic agonists, potentially facilitating splanchnic venous emptying and physiological autotransfusion.

Despite its distinct mechanism of action, centhaquine did not produce a significant change in RVR, suggesting that venous return from the systemic circulation to the right heart was largely maintained. This observation may reflect several factors, including redistribution of blood among vascular beds, venous distension resulting from increased venous volume (Guyton et al., 1958), the limited effect of selective venoconstriction on RVR due to the relatively thin muscular layer of veins, and potential reductions in blood viscosity following crystalloid infusion (Jansen et al., 2010). Additionally, the irregular geometry of the venous wall, which tends to assume a more rounded lumen during constriction, may reduce the surface area in contact with flowing blood, thereby mitigating any rise in RVR (Gelman, 2008; Jacobsohn et al., 1997; Pang, 2001; Rothe, 1993).

In our spontaneously breathing patients (i.e., those with consistently negative pleural pressures), estimated CVP values remained low and near atmospheric pressure throughout the 300‐min observation period. Given the stability of respiratory parameters, pleural pressure likely exhibited minimal temporal variation, suggesting that right atrial filling conditions remained relatively stable during the study period (Vieillard‐Baron et al., 2016). Additionally, physiological limits to inspiratory augmentation of venous return – such as venous collapse – restrict further flow increases, as reflected by the plateau in the venous return curve (Figure 1) (Magder, 2018; Permutt & Riley, 1963), particularly in hypovolaemic individuals. Accordingly, the observed increase in CO was physiologically consistent with recruitment of stressed venous volume and augmentation of venous return. The persistence of near‐atmospheric estimated CVP values despite increasing CO challenges the conventional view that venous return rises solely in response to decreased CVP and may reflect enhanced reciprocal volume redistribution among compliant vascular compartments, generating a pressure gradient largely governed by flow‐dependent dynamics rather than exclusively by the classical VRdP (Bendjelid, 2005; Levy, 1979).

Equally notable, the observed increases in venous return and CO were not accompanied by haemodynamic evidence of adverse right‐sided circulatory effects during the study period. Although pulmonary haemodynamics were not directly assessed, respiratory parameters remained stable throughout the study, suggesting preservation of effective heart–lung interactions and the absence of clinically apparent flow limitation (Lopez‐Muniz et al., 1968; Permutt et al., 1962; West et al., 1964). These conditions are physiologically compatible with preserved pulmonary blood flow and left ventricular filling during spontaneous breathing under predominantly zone III conditions (Magder, 2018; West et al., 1964).

The observed increase in MAP over time, in the context of stable SVR, increased *C*_art_, and unchanged *E*_a_, is indicative of enhanced venous return and stroke volume. Collectively, these findings suggest improved mechano‐energetic efficiency in ventriculo‐arterial coupling. Activation of central α_2A_‐adrenoreceptors by centhaquine reduces sympathetic outflow and left ventricular afterload (Chalkias et al., 2024), thereby facilitating forward flow and systemic haemodynamics. By contrast, exogenous catecholamines may exacerbate arteriolar constriction and tissue ischaemia during circulatory shock (De Backer & Foulon, 2019).

Based on its mechanistic profile and the present findings, centhaquine may support arterio‐venous coupling and preserve or improve the critical closing pressure‐to‐*P*_mcf_ gradient, potentially independent of changes in CO. This relationship is physiologically plausible yet complex, warranting further mechanistic investigation. Notably, we recently described a novel intravascular volume at zero transmural pressure, termed ‘rest volume’ (*V*_r_), which appears to serve two principal functions under steady‐state conditions: preventing increases in RVR and maintaining critical closing pressure (Chalkias, 2023b; Chalkias, Laou, Papagiannakis et al., 2022). Future studies should evaluate centhaquine's effects on *V*_r_ and arterio‐venous coupling in MHVS and other shock states.

The dissociation between changes in arterial pressure and calculated SVR observed in the present study highlights the known limitations of SVR as a simplified descriptor of vascular tone under dynamic physiological conditions (Maas, de Wilde et al., 2012). Accordingly, direct measurements of *R*_ven_/*R*_art_ may provide more accurate assessments of systemic vasomotor tone under dynamic conditions such as MHVS.

This study presents a physiological analysis nested within a multicentre, open‐label, single‐arm phase IV trial and should be interpreted within this framework. The sample size, limited by the intensive physiological characterisation required, is appropriate for mechanistic hypothesis testing but constrains statistical power and precludes causal inference regarding clinical outcomes. Nevertheless, patients were randomly selected by an independent individual. Although exploratory, the observed temporal consistency across multiple haemodynamic variables supports the internal coherence of the physiological findings.

The study population primarily comprised young to middle‐aged adults with MHVS who were spontaneously breathing and had preserved cardiac function. Therefore, extrapolation to elderly patients, mechanically ventilated individuals, or shock states dominated by myocardial dysfunction or severe vasoplegia should be approached with caution. Short‐term follow‐up similarly limits broader interpretation, though the pragmatic, real‐world study design enhances clinical relevance. The modest fluid administration (∼135 mL h^−1^) minimises confounding by volume effects, yet the potential influences of passive venous recoil, compartmental fluid shifts and spontaneous inspiratory effort were not directly assessed. Additionally, the absence of a control group precludes determination of whether the observed haemodynamic changes differed from those attributable to standard resuscitation alone. Nevertheless, the findings of the present study are supported by prior experimental and clinical investigations demonstrating similar haemodynamic effects (Chalkias et al., 2024; Gulati et al., 1991; Lavhale et al., 2013; Srimal et al., 1990).

Several haemodynamic variables in the present study were model‐derived rather than directly measured, including SVR, CVP, *P*_mca_, RVR and efficiency indices. Consequently, propagation of estimation error across interconnected variables cannot be excluded. In addition, MAP was assessed non‐invasively using oscillometric cuff measurements rather than by indwelling arterial catheterisation. Although invasive arterial monitoring is traditionally considered the reference standard in shock, non‐invasive oscillometric measurements of MAP are reasonably accurate at the population level and are often more reliable for MAP than for systolic pressure, even in hypotensive states (Nedel et al., 2022; Seidlerová et al., 2019).

Importantly, modern non‐invasive monitoring devices have been shown to accurately detect low MAP – a critical determinant of tissue perfusion – as well as changes in MAP induced by therapeutic interventions, including in patients receiving vasopressors and those with cardiac arrhythmias (Lakhal et al., 2009, 2012, 2015). While absolute values may differ modestly from invasive measurements, directional changes and temporal trends are generally preserved. In the present study, MAP served as a complementary variable within an integrative physiological framework focused on venous return, CO and pressure–flow relationships; thus, the principal mechanistic findings are not dependent on high‐fidelity arterial waveform analysis.

Arterial pressure was measured using the same device, technique, and protocol at all time points, supporting internal validity and within‐subject comparisons. Furthermore, patients were spontaneously breathing and evaluated early in shock, a clinical context in which routine arterial cannulation is not always indicated. Consistent with recent ESICM guidelines (Monnet et al., 2025), which recommend arterial catheterisation primarily in patients with shock that is refractory to initial therapy, the observed haemodynamic response suggested responsiveness to centhaquine and fluid administration. Accordingly, invasive arterial monitoring was not mandated by guideline‐based practice in this clinical context. In line with recent high‐quality evidence demonstrating that non‐invasive blood‐pressure monitoring is non‐inferior to early arterial catheterisation in patients with shock (Muller et al., 2025), the observed increase in MAP should be interpreted as a supportive, convergent finding rather than a standalone endpoint. Nevertheless, the absence of invasive arterial monitoring remains a limitation when interpreting absolute MAP values.

Despite these limitations, this study provides the first comprehensive physiological assessment of early selective venoconstriction in spontaneously breathing patients with mixed shock (Figure 7), an area not previously explored. It offers mechanistic insight that may explain prior clinical observations (Chalkias, 2023a; Chalkias et al., 2024; Gulati, Choudhuri et al., 2021; Gulati, Jain et al., 2021; Sondergaard et al., 2019), although the long‐term effects of centhaquine‐mediated selective venoconstriction in circulatory shock require further evaluation. While centhaquine is designed to minimise cardiovascular adverse effects, future studies should systematically assess long‐term cardiovascular safety, particularly in vulnerable populations. Similarly, investigations into renal perfusion are warranted, as initial haemodynamic improvements may not predict long‐term renal recovery or prevention of acute kidney injury. Given centhaquine's selective α_2_‐adrenoreceptor activity, its balanced cardiovascular profile may favour renal vasodilation and functional restoration (Chalkias, 2025). Moreover, given that shock‐induced cerebral hypoperfusion contributes to neurological sequelae, the potential impact of centhaquine on cognitive and neurological recovery following prolonged shock warrants further investigation. Ultimately, long‐term mortality remains the critical endpoint, emphasising the need for large‐scale, randomised controlled trials with extended follow‐up to determine whether centhaquine improves survival in circulatory shock.

**FIGURE 7:**
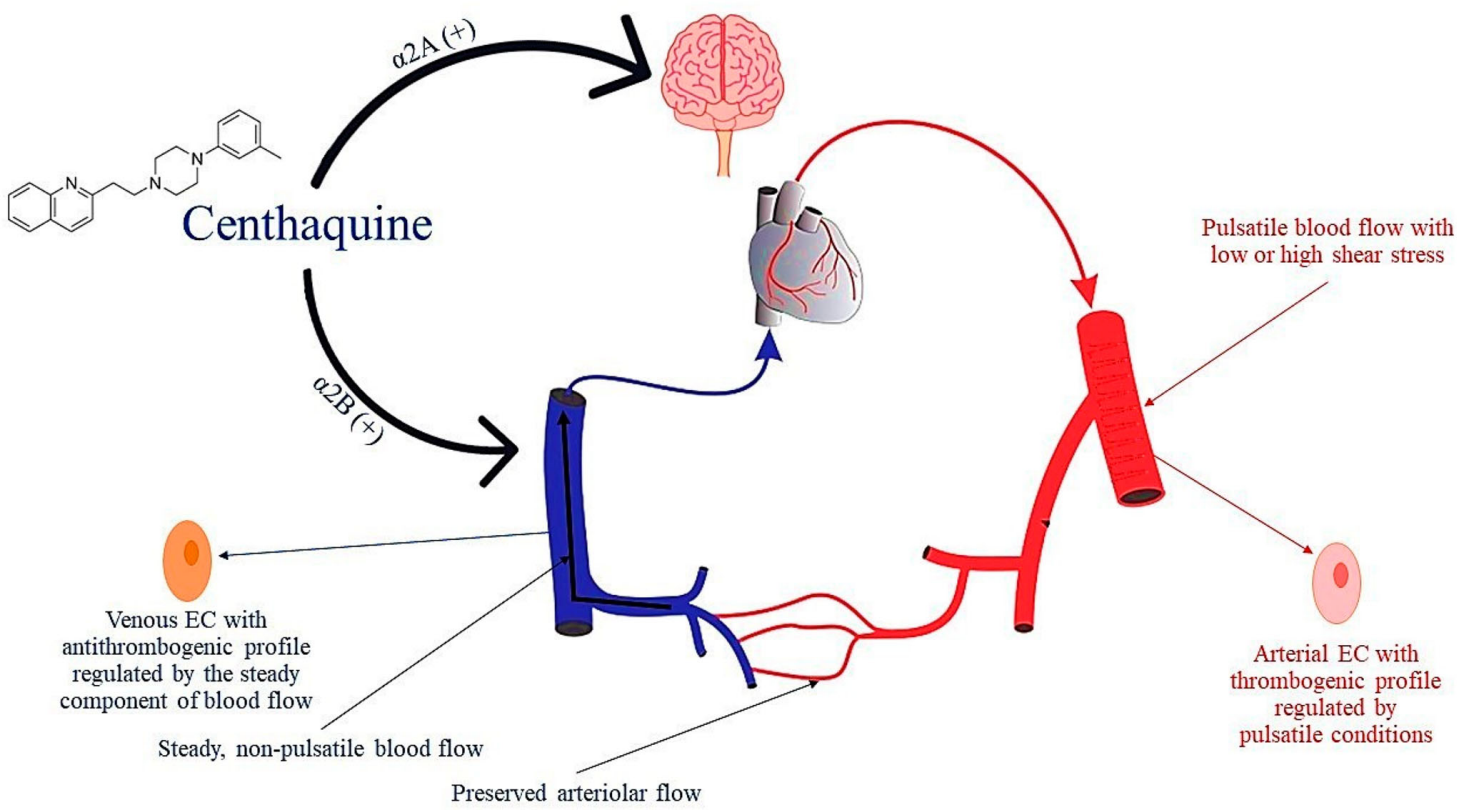
Selective venoconstriction with centhaquine. Activation of α_2B_‐adrenoreceptors promotes venous constriction and augments venous return, while central α_2A_‐adrenoreceptor activation may attenuate sympathetic outflow, thereby supporting forward flow and systemic haemodynamics. EC, endothelial cells. Adapted from Chalkias (2023a).

In this exploratory physiological study, centhaquine administration was associated with increases in *P*_mca_, VRdP, CO and arterial pressure, without increases in RVR or *E*_a_, while fluid administration remained modest throughout the study period. These findings support further investigation of selective venoconstriction as a physiological strategy to augment venous return during shock resuscitation.

## AUTHOR CONTRIBUTIONS

Conceptualisation: Athanasios Chalkias, Anil Gulati. Data curation: Athanasios Chalkias, Anil Gulati, Aman Khanna, Dharmesh Shah. Formal analysis: Athanasios Chalkias. Investigation: Aman Khanna, Krish Vaidya. Methodology: Athanasios Chalkias. Project administration: Aman Khanna, Dharmesh Shah. Visualisation: Athanasios Chalkias. Writing – original draft: Athanasios Chalkias. Writing – review and editing: Athanasios Chalkias, Aman Khanna, Krish Vaidya, Dharmesh Shah, Anil Gulati. All authors have read and approved the final version of this manuscript and agree to be accountable for all aspects of the work in ensuring that questions related to the accuracy or integrity of any part of the work are appropriately investigated and resolved. All persons designated as authors qualify for authorship, and all those who qualify for authorship are listed.

## CONFLICT OF INTEREST

Dharmesh Shah is an employee of Pharmazz India Pvt Ltd, India. Anil Gulati is an employee of Pharmazz, Inc., Willowbrook, IL, USA, and holds issued and pending patents related to this study. Athanasios Chalkias, Aman Khanna and Krish Vaidya declare that they have no competing interests.

## FUNDING INFORMATION

This research received no external funding.

## GENERATIVE AI STATEMENT

No generative artificial intelligence (AI) tools or AI‐assisted technologies were used in the preparation, writing, revision, analysis, interpretation or editing of this manuscript.

## Data Availability

Data can be made available upon request after publication through a collaborative process. Researchers should provide a methodologically sound proposal with specific objectives in an approval proposal. Please contact the corresponding author for more information.
